# Mitochondria Dysfunction and Cell Apoptosis Limit Resistance of Nile Tilapia (*Oreochromis niloticus*) to Lethal Cold Stress

**DOI:** 10.3390/ani12182382

**Published:** 2022-09-12

**Authors:** Ran Liu, Renyan Liu, Guili Song, Qing Li, Zongbin Cui, Yong Long

**Affiliations:** 1 College of Fisheries and Life Science, Dalian Ocean University, Dalian 116023, China; 2 State Key Laboratory of Freshwater Ecology and Biotechnology, Institute of Hydrobiology, Chinese Academy of Sciences, Wuhan 430072, China; 3 College of Advanced Agricultural Sciences, University of Chinese Academy of Sciences, Beijing 100049, China; 4 Guangdong Provincial Key Laboratory of Microbial Culture Collection and Application, State Key Laboratory of Applied Microbiology Southern China, Institute of Microbiology, Guangdong Academy of Sciences, Guangzhou 510070, China

**Keywords:** cell apoptosis, cold stress, mitochondria dysfunction, Nile tilapia, tissue damage

## Abstract

**Simple Summary:**

Sensitivity of Nile tilapia (*Oreochromis niloticus*) to cold stress represents a major concern for both aquaculture and theoretical study; however, the cellular and molecular mechanisms determining cold susceptibility of it remain largely unknown. In this study, we first estimated the median survival time of juvenile Nile tilapia under exposure to lethal cold stress (12 °C). The fish were classified as cold-sensitive or cold-tolerant based on their behavioral manifestation after exposed to 12 °C for 3 days. Subsequently, histological, biochemical and gene expression analyses were performed for the fish with different cold resistance to explore the cellular and molecular events underlying cold susceptibility of Nile tilapia. We found that exposure of Nile tilapia to lethal cold stress caused systemic tissue structure changes, mitochondrial swelling and dysfunction, induction of apoptosis and endoplasmic reticulum (ER) stress-related genes and cell apoptosis. The extent of these adverse cellular and molecular events determines an individual’s ability to survive cold stress. Our data indicate that mitochondria dysfunction and mitochondria-mediated cell apoptosis are the main factors limiting Nile tilapia’s cold resistance.

**Abstract:**

Inability of Nile tilapia (*Oreochromis niloticus*) to withstand cold stress represents a major economic concern, which restricts the culture area, limits the growing period and even results in mass mortality in cold seasons. However, the cellular and molecular mechanisms determining cold susceptibility of Nile tilapia remain largely unknown. In this study, we characterized the ability of juvenile Nile tilapia to survive lethal cold stress (12 °C) and the median survival time (LT50) of the experimental fish under exposure to 12 °C cold stress was estimated as 3.14 d. After being exposed to 12 °C for 3 d, the survivors that lost equilibrium (LE) and those that swam normally (NO) were regarded as cold-sensitive and cold-tolerant, respectively. The untreated (Ctrl), NO and LE fish were subjected to histological, biochemical and gene expression analyses to explore the cellular and molecular events underlying cold susceptibility of Nile tilapia. Exposure of Nile tilapia to lethal cold stress caused systemic tissue structure changes, mitochondrial swelling and dysfunction, induction of apoptosis and endoplasmic reticulum (ER) stress-related genes and cell apoptosis. The extent of these adverse cellular and molecular events determines an individual’s ability to survive cold stress. Our data indicate that mitochondria dysfunction and mitochondria-mediated cell apoptosis are the main factors limiting Nile tilapia’s cold resistance.

## 1. Introduction

Tilapia is a group of fish with great economic importance. The global production of Nile tilapia, one of the most cultivated tilapiine species, exceeded 4.4 million tons in 2020, accounting for 9% of the total production of finfish in inland aquaculture [1]. Nile tilapia originate from tropical, subtropical Africa and the Middle East, and are widely distributed in Nile and Niger River basins and in lakes Tanganyika, Albert, Edward and George [2]. The water temperature of the native habitats for tilapia species ranges from 28 °C to 34 °C in warm seasons and from 22 °C to 26 °C in cold seasons; the lowest temperature that tilapia may encounter in the natural habitat is 17 °C to 24 °C in high-altitude lakes [2,3]. Due to the warm environment during evolution, tilapia fishes become cold-sensitive, which stop growth and feeding below 22 °C and 20 °C, respectively [4], and generally die within a few days when exposed to low temperatures of 10 °C to 12 °C [5]. The lowest winter temperature in the main culturing zone for tilapia, such as in China, is readily below the thermal minimum of tilapia.

The susceptibility of tilapia to low temperatures represents a major economic concern, which limits growing seasons and leads to mass over-winter mortality [6]. Except for estimating the cold resistance of different selected species or strains [6,7,8], the mechanisms determining cold resistance (or susceptibility) of tilapia have attracted intensive research interest. Histological investigations revealed that exposure of Nile tilapia to acute cold stress (13 °C) resulted in pathological tissue structure changes, such as vacuolation of neuropil and curling of secondary gill lamellae [9]. Cold-induced changes to blood biochemical parameters of tilapia were also characterized, which reflect the status of body health. Exposure to cold stress markedly decreased activities of antioxidant enzymes such as superoxide dismutase and catalase [10,11,12], indicating impairment of antioxidant functions. Nile tilapia exposed to cold temperature demonstrated significantly decreased concentrations of glucose and triglyceride in the serum [11,13], suggesting depletion of energy deposition. Furthermore, exposure of tilapia to cold stress increased activities of serum aspartate aminotransferase, alanine aminotransferase and lactate dehydrogenase [11] and concentrations of urea and uric acid [14], indicating the occurrence of tissue damage. 

Genetic factors were reported to play dominant functions in determining cold resistance of tilapia based on variations in the ability of different species and their hybrids to survive lethal cold stress [10,15]. Comparative transcriptomic studies for species and strains with distinct cold resistance were conducted to explore the genetic pathways underlying the cold resistance of tilapia. Biological pathways including FoxO signaling and metabolic regulation were suggested to account for the differential low-temperature limit between Nile tilapia and zebrafish (*Danio rerio*) [16]. The cold-tolerant strain of blue tilapia (*Oreochromis aureus*) demonstrated downregulation of biological processes including glycolysis/gluconeogenesis (in gills) and amino-acid biosynthesis (in liver) upon cold stress in comparison with the cold-sensitive strain [17]. 

Cold-responsive genes (CRGs) in tissues such as the liver and kidney of Nile tilapia were identified through RNA sequencing and subjected to functional enrichment analysis to shed light on the biological functions influenced by cold stress. The CRGs of Nile tilapia liver were highly enriched in biological processes such as nucleic acid synthesis and metabolism, apoptosis and immunity [18], while those of the kidney were mainly associated with metabolism and immunity [14]. Cold-induced alternative splicing in tissues including testis, brain and kidney of Nile tilapia were characterized and many differentially spliced genes were found to be involved in the circadian clock pathway [19]. Small RNA sequencing was conducted to identify cold-regulated miRNAs in the head kidney of Nile tilapia and the predicted target genes of the cold-responsive miRNAs are mainly associated with lipid metabolic processes and response to temperature stimulus [12]. 

The previous studies have expanded our understanding of the cold-elicited adverse effects and the genetic programs associated with cold resistance of tilapia; however, the cellular and molecular mechanisms underlying cold susceptibility of tilapia remain to be further investigated. The cellular events and molecular pathways leading to tissue dysfunction upon severe cold stress are largely unknown. In this study, we characterized the cold-resistance ability of juvenile Nile tilapia and classified the individuals as cold-tolerant and cold-sensitive based on their capability to withstand lethal cold exposure. Histological analyses were performed for multiple tissues from the individuals with different cold resilience. We found that mitochondrial swelling was the most prominent subcellular change caused by exposure to lethal cold stress. Subsequent experiments proved that hardiness of mitochondrial function, expression of apoptosis and ER stress-associated genes are intimately related to cold resilience of Nile tilapia. Our data suggest that mitochondria dysfunction and mitochondria-mediated cell apoptosis limit resistance of Nile tilapia to lethal cold stress.

## 2. Materials and Methods

### 2.1. Fish Maintenance

Larvae of Nile tilapia were obtained from the Freshwater Fisheries Research Center, Chinese Academy of Fishery Sciences (Wuxi, China). The fish were raised in aquariums supplied with recycling water, as previously described [20]. Water temperature was maintained at 28 ± 0.5 °C and the fish room was illuminated with 12 h/12 h light cycles (from 8 AM to 8 PM). Water quality parameters including pH, dissolved oxygen and ammonia were measured as 7.0–7.5, >5 mg/L and <0.01 mg/L, respectively. The fish at larval stage were fed with nauplii of brine shrimp and the fingerlings were fed with a commercial floating fish feed (crude protein ≥ 36%, crude fats ≥ 5.0%, crude fiber ≤ 4.0% and ash ≤ 14%). The fish were fed twice daily to satiation. Three- to six-month-old fish were used for the experiments. 

### 2.2. Cold Treatment of Nile Tilapia

**Figure 1:**
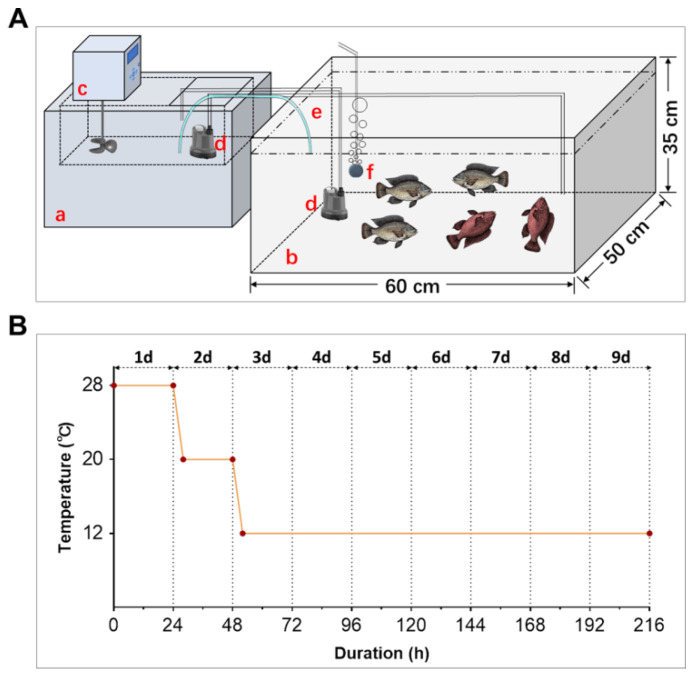
The facilities used for cold exposure and temperature treatment regime. (**A**) Diagram demonstrating the equipment used for cold exposure. A PC200 A40 ARCTIC Refrigerated Circulator from Thermo Scientific was used for temperature control and the fish were held in the customized plastic box. Components: a, refrigerated circulator; b, customized plastic box; c, controller of the circulator; d, mini pumps (40 W, 2000 L/h); e, siphon pipe; f, air stone. The fish that lost equilibrium are shown in red. (**B**) Line chart illustrating the gradient temperature regime for cold exposure. The red dots indicate the beginning and ending time of treatment for each temperature.

The facilities used for cold exposure are shown in Figure 1A, consisting of a PC200 A40 ARCTIC Refrigerated Circulator from Thermo Scientific (Waltham, MA, USA) and a customized plastic box. Two mini pumps were used for water circulation between chambers of the refrigerated circulator and the plastic box. A siphon pipe was used for water volume balance. The water was aerated continuously using an air stone connected to an air pump. The experimental fish were held in the plastic box (filled with 100 L water). 

The fish were fasted for 24 h before being transferred into the exposure box. They were acclimated to the experimental condition at 28 °C for 24 h. After that, the temperature was decreased from 28 °C to 20 °C (2 °C/h) and maintained for 20 h. The temperature was further decreased to 12 °C (2 °C/h) and then maintained for the remaining part of the experiment (Figure 1B). The fish were not fed and checked every 12 h during cold exposure. Death was judged by a lack of response to agitation with a thin stick. Death was further ensured by placing the dead fish into water of normal temperature where none of them could revive. The dead fish were removed and subjected to standard length and body weight measurements. Survival time of the fish at 12 °C was used as the index for cold resistance. The experiment was repeated 3 times and 20 fish were included in each treatment. 

During the experiments, we found that the cold-injured fish usually lost equilibrium (LE, Figure 1A, shown in red) at 12 to 24 h before death. At a given time point, the individuals with LE symptoms couldn’t survive when returned to normal temperature (28 °C), while those that swam normally (NO) could revive. This indicated that the LE fish were more susceptible to cold stress and had higher extent of cellular and tissue damages than the NO ones. To explore the cellular and molecular mechanisms underlying cold susceptibility of Nile tilapia, the fish were treated at 12 °C for 72 h and the survivors were classified as NO and LE, respectively. Samples collected from the untreated (Ctrl), NO and LE fish were subjected to subsequent histological, biochemical and gene expression analyses. 

### 2.3. Histological Analysis

Histological analysis was conducted to investigate the effects of cold exposure on tissue structure. The fish were exposed to cold stress as described above. After cold exposure, the fish were euthanized by immersion into ice-slurry for 3 min as previously described [21]. The fish were dissected and tissues including brain (cerebellum), gill, heart, kidney, liver, muscle and spleen were collected. The samples were fixed in 4% PFA (Beyotime Biotechnology, Shanghai, China) at 4 °C overnight. After fixation, the samples were dehydrated with alcohol gradients, embedded with paraffin and sectioned into 4 μm slices. The slices were stuck to glass slides, soaked in xylene for dewaxing and rehydrated in ethanol gradients. Finally, the slides were subjected to H&E staining. Photographs of the tissue sections were taken using an Aperio VERSA Brightfield, Fluorescence & FISH Digital Pathology Scanner from Leica (Wetzlar, Germany). Subjective analyses were performed for the photographs to identify changes of tissue structure among the samples from different experimental groups. The photographs were examined independently by different investigators to avoid personal preconceptions when making their judgments.

### 2.4. Transmission Electron Microscopy

Transmission electron microscopy was performed as previously reported [22] to investigate effects of cold exposure on the subcellular structure of the brain (cerebellum) and liver. After ultrathin sectioning and staining, photographs of the sections were taken using a HT7700 transmission electron microscope (Hitachi High-Tech, Tokyo, Japan). The photographs were analyzed using ImageJ2 [23] to measure mitochondria size (the largest distance between the ends of mitochondria).

### 2.5. Mitochondrial Membrane Potential Measurement

Brain and liver were collected as described above. To measure mitochondrial membrane potential (MMP), mitochondria were isolated from the samples using the Tissue Mitochondria Isolation Kit (Beyotime, Shanghai, China) according to the manufacturer’s instructions. The isolated mitochondria were suspended with 180 μL PBS (pH 7.4) in 1.5 mL centrifuge tubes. After that, 20 μL JC-1 working solution (obtained from Beyotime, Shanghai, China) was added into the mitochondria suspension. The samples were mixed thoroughly by gently flicking the tubes. Flow cytometry was performed using a CytoFLEX S Flow Cytometry (Beckman Coulter, Brea, CA, USA).

### 2.6. ATP Concentration Measurement

ATP content of the brain and liver tissues was analyzed using the Enhanced ATP Assay Kit (Beyotime, Shanghai, China) following the manufacturer’s instructions. Briefly, the tissue homogenate was centrifuged at 4 °C, 12,000 rpm for 5 min. After centrifugation, the supernatant was collected and mixed with the ATP detection reagent. Fluorescence was measured using a GloMax^®^ 20/20 Luminometer (Promega Biotech, San Luis Obispo, CA, USA). A standard curve was generated using the ATP solution included in the kit. Protein concentration of the samples was measured using the Enhanced BCA Protein Assay Kit (Beyotime, Shanghai, China). Six biological replicates for each group were included in the assay. ATP concentration was normalized to the amount of total protein (nM/mg protein). 

### 2.7. Total RNA Extraction and Quantitative Real-Time PCR

Quantitative real-time PCR (qPCR) was performed as previously reported to analyze gene expression [22]. Brain and liver were collected and subjected to total RNA extraction using TRIzol Reagent (Invitrogen, Waltham, MA, USA). The samples were homogenized in TRIzol using a LyserPro Procedural Cryogenic Tissue Grinder from Monad (Shanghai, China). Concentration of total RNA was measured using a Q5000 UV–Vis Spectrophotometer (Quawell, Fremont, CA, USA) and quality of RNA was examined by agarose gel electrophoresis. One microgram of total RNA was used for synthesis of the first-strand cDNA using the TransScript® All-in-One All-in-one First Strand cDNA synthesis SuperMix from TransGen (Beijing, China). The primers used for qPCR assays were designed using the online software Primer3Plus (www.primer3plus.com, assessed on 12 September 2022). The sequence, amplicon size and efficiency of the qPCR primers are listed in Table 1. A CFX Duet Real-Time PCR System (Bio-Rad, Hercules, CA, USA) was used for qPCR assays. The reagents, program and method of data analysis were the same as the previous study [22]. Expression of two commonly used internal references, *ef1α* and *gapdh,* were analyzed and the geometric average of their relative expression was used to normalize expression of the target genes. 

### 2.8. TUNEL Assay

TUNEL (Terminal deoxynucleotidyl transferase dUTP nick end labeling) assay was conducted for brain and liver to assess the effects of cold exposure on cell apoptosis. The samples were collected, fixed, embedded and sectioned using the same protocol as histological analysis. The sections were deparaffinized in xylene and rehydrated in gradient ethanol solutions. After rehydration, the sections were treated with a proteinase K solution (obtained from Servicebio Technology Co., Ltd., Wuhan, China) for 25 min at 37 °C. Then, the slides were thoroughly washed using PBS (pH 7.4) and the reaction solution contained in the One Step TUNEL Apoptosis Assay Kit (Beyotime, Shanghai, China) was added onto the sections. The sections were placed into a flat wet box and incubated at 37 °C for 2 h. After being washed 3 times with PBS (pH 7.4), the sections were stained using DAPI solution at room temperature for 10 min. Subsequently, the slides were mounted and observed using a Leica SP8 confocal microscope (Wetzlar, Germany). Photos for the sections were taken and analyzed with ImageJ2 [23] for quantification of TUNEL signal (fluorescence intensity).

### 2.9. Statistical Analysis

Statistical analysis was performed using IBM SPSS statistics version 25 (Armonk, NY, USA). The correlation between survival time and variables including standard length, body weight and conditional factor was analyzed by bivariate Pearson correlation. Median survival time (LT50) of Nile tilapia upon 12 °C cold stress was estimated by analyzing the data of survival time and average death rate through Probit regression. One-way analysis of variance (ANOVA) followed by multiple comparisons was performed to test significance of difference among the experimental groups. Significant difference (*p* < 0.05) between means of the treatment groups was marked using different letters.

## 3. Results

### 3.1. Survival Time of Nile Tilapia under Acute Cold Stress

**Figure 2:**
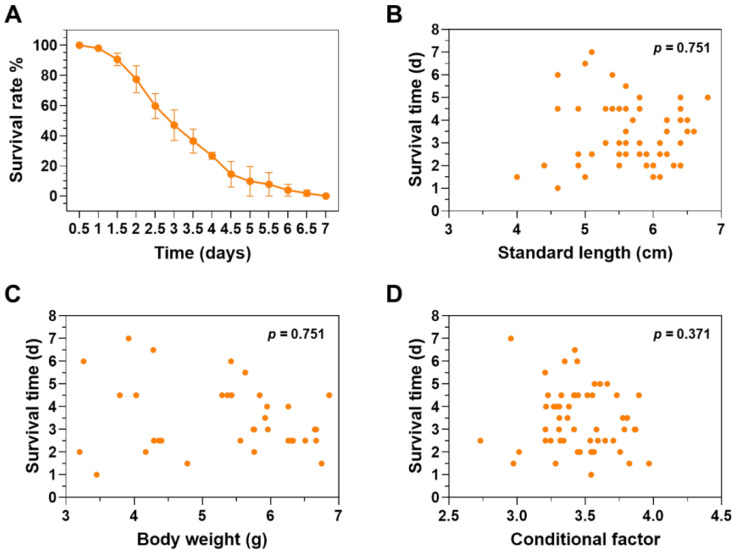
Survival time of the experimental fish under lethal cold stress. (**A**) Survival time of the individuals under 12 °C lethal cold stress. The error bars represent standard deviation (SD, *n* = 3). (**B**–**D**) There is no significant correlation between survival time and standard length (**B**), body weight (**C**) and conditional factor (**D**) of the experimental fish.

The experimental fish were exposed to gradient low-temperature stress to evaluate cold resistance. Mortality of the fish began at 1 d and all the fish died at 7 d after the water temperature was decreased to 12 °C (Figure 2A). Median survival time (LT50) of the fish upon exposure to 12 °C cold stress was estimated as 3.14 d (the 95% confidence interval was 2.96–3.31 d). No significant correlation was identified between survival time and standard length (Figure 2B), body weight (Figure 2C) and conditional factor (Figure 2D), indicating that body size and nutritional status of the experimental fish had no significant effect on resilience against lethal cold stress.

### 3.2. Exposure of Nile Tilapia to Cold Stress Caused Extensive Tissue Damage

**Figure 3:**
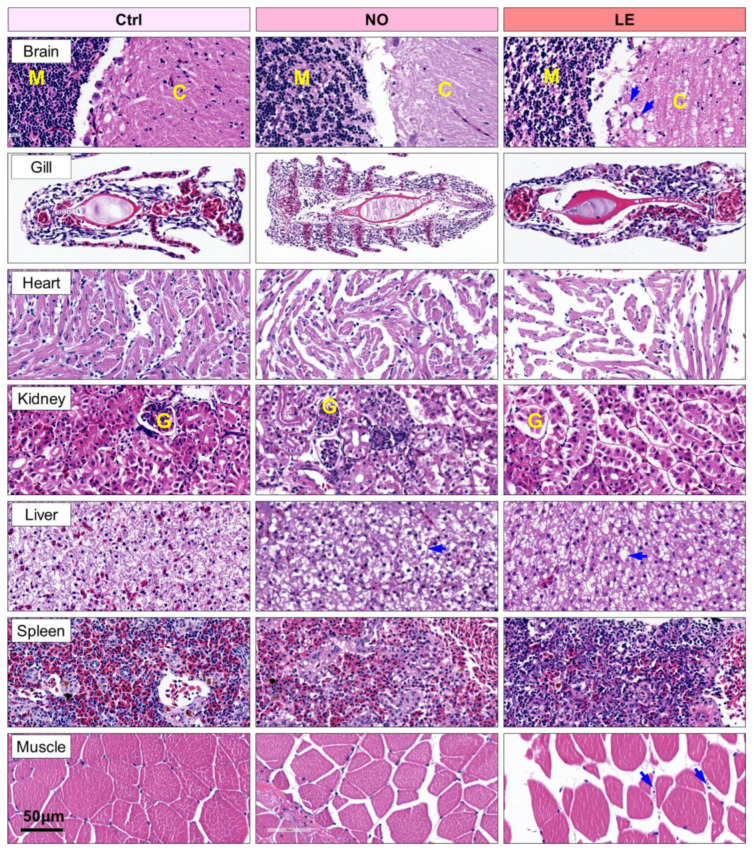
Exposure of Nile tilapia to lethal cold stress caused extensive tissue damage. After being held at 12 °C for 72 h, both the normal fish (NO, individuals that could swim normally at the sampling time) and those lost equilibrium (LE) were sampled. Ctrl indicates fish not exposed to cold stress. Sample numbers for the Ctrl, NO and LE groups are as follows: brain 1, 2, 2; gill 3, 4, 4; heart 3, 4, 4; kidney 1, 2, 2; liver 1, 2, 2; muscle 3, 4, 4; spleen 1, 2, 2. Representative photos are shown. The blue arrows in the brain and liver sections indicate vacuoles, while those in the muscle sections indicate necrotic fibers. M, medulla; C, cortex; F, gill filament; G, glomerulus.

To characterize the effects of exposure to lethal cold stress on tissue structure, tissues including brain (cerebellum), gill, heart, kidney, liver, muscle and spleen were collected from the Ctrl, NO and LE fish and subjected to histological analyses. Exposure to lethal cold stress resulted in severe damage to all the characterized tissues. All the sampled fish from the same group demonstrated identical changes in the same organs; representative photographs are shown in Figure 3. Consistent with their stronger resistance to cold stress, the NO fish demonstrated milder tissue damage in comparison with the LE ones (Figure 3). 

After cold exposure, the brain demonstrated obvious vacuoles (indicated by blue arrows) in both the medulla and cortex; nuclei density in medulla of both the NO and LE sections was markedly decreased in comparison with that of the Ctrl (Figure 3). Epithelia hyperplasia and lamella collapse were identified for gill filaments of the NO and LE fish, respectively (Figure 3), demonstrating that cold exposure impaired respiration function of Nile tilapia. The size of heart muscle fibers was reduced and the space between the fibers was enlarged (Figure 3), indicating that exposure to acute cold stress resulted in atrophy of cardiac muscle fibers. For the kidney, the number of cells in the glomerulus decreased and the tubule epithelial cells shrunk (Figure 3). A large number of vacuoles (indicated by blue arrows) could be found in the liver sections of the cold-treated fish; the density of vacuoles in sections of the NO fish was comparable to those of the LE samples, suggesting that liver is highly susceptible to acute cold stress (Figure 3). Cytoplasm of the red blood cells in the spleen sections was heavily stained with eosin (dark red); cold exposure significantly reduced the number of red blood cells in the spleen sections, and the red blood cells also shrunk (Figure 3). Finally, cold exposure resulted in features of atrophy in skeletal muscle, including the reduction in muscle fiber diameter and appearance of necrotic fibers (blue arrows, Figure 3).

Together, these results indicate that exposure to acute cold stress caused systemic injury to the tissues of Nile tilapia and the extent of tissue damage was in accordance with the individual’s resistance to cold stress.

### 3.3. Cold Exposure Caused Mitochondrial Swelling

After revealing that cold exposure could cause extensive tissue damage in Nile tilapia, we further performed transmission electron microscopy to characterize the adverse impacts of cold stress on cell organelles. Brain and liver were subjected to transmission electron microscopy because loss of equilibrium was the most prominent symptom distinguishing the individuals with different ability to endure acute cold stress (distinct between the LE and NO groups) and liver was found to be more sensitive to cold stress than other organs (Figure 3). 

**Figure 4:**
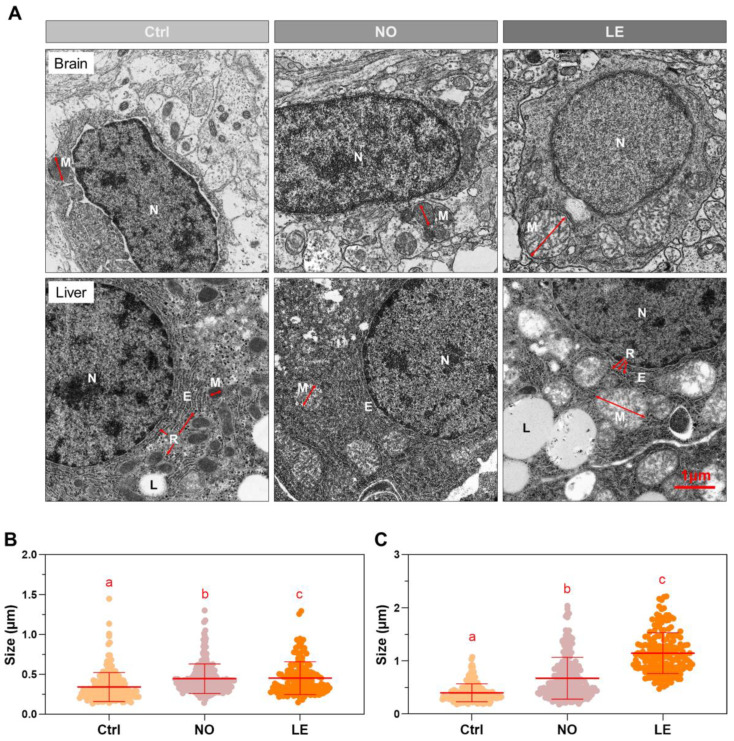
Transmission electron microscopy for brain and liver of Nile tilapia exposed to lethal cold stress. (**A**) Transmission electron microscopy photographs indicating subcellular changes caused by cold exposure. Sample numbers for the Ctrl, NO and LE groups are as follows: brain 2, 2, 2; liver 2, 2, 2. Representative photos are shown. M, mitochondria; N, nucleus; E, endoplasmic reticulum; L, lipid droplet; R, ribosome. The double-headed arrows demonstrate the distance measured as mitochondria size. (**B**,**C**) Size of mitochondria measured from photographs of the brain (**B**) and liver (**C**) sections. All data points are shown (*n* = 165 to 241) and the lines over the data points represent mean and SD. Different letters above the data sets indicate significant difference between the means (*p* ≤ 0.05).

The most obvious subcellular change found for both the brain and liver of the fish exposed to cold stress was mitochondrial swelling. Mitochondria of the Ctrl sections were optically condenser than those of the NO and LE samples (Figure 4A). Furthermore, cold exposure also resulted in disturbance to the endoplasmic reticulum (ER) structure in liver; regular arrangement of ER and ribosomes in the Ctrl sections was disrupted in both the NO and LE samples (Figure 4A). The ultrathin sections were subjected to mitochondria size measurements to provide an index for assessing the degree of mitochondria structure disruption. The LE samples had the largest average mitochondria size in both brain and liver. The NO fish also demonstrated significantly larger mean mitochondria size than the untreated controls (Figure 4B,C). These data indicate that mitochondria are the main subcellular targets impacted by cold stress and the extent of mitochondria structure disruption is related to an individual’s cold endurance.

### 3.4. Cold Exposure Resulted in Mitochondria Dysfunction

**Figure 5:**
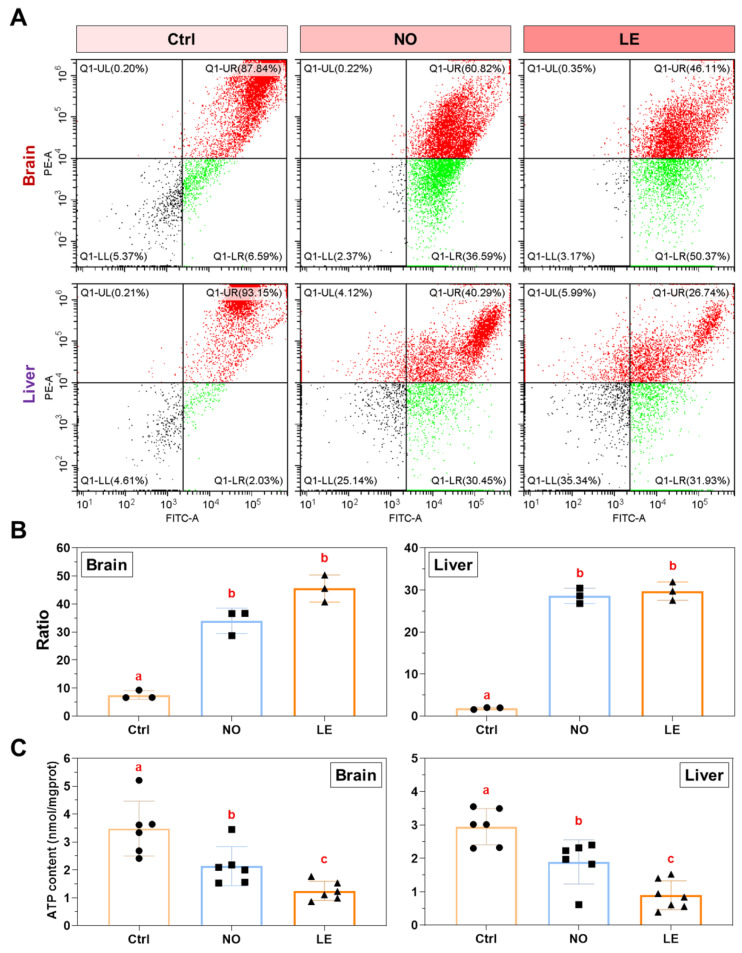
Mitochondrial membrane potential and tissue ATP content. (**A**) Measurement of mitochondria membrane potential (MMP) by JC-1 staining and flow cytometry. (**B**) Ratio of green/red fluorescence, which reflects change in mitochondria membrane potential. (**C**) Tissue ATP concentration. The error bars represent SD (*n* = 3). The dots, squares and triangles represent data of the corresponding experimental groups shown in the horizontal axis. Different letters above the error bars indicate significant difference between the means (*p* ≤ 0.05).

Since exposure to cold stress caused disturbance to the mitochondria structure of Nile tilapia cells, it was probable that the functions of mitochondria were also impaired. Mitochondrial membrane potential (MMP) assays based on JC-1 staining and flow cytometry were conducted to evaluate effects of cold exposure on mitochondrial activity. The results indicate that mitochondria isolated from the whole brain and liver of the NO and LE fish demonstrated significantly lower MMP in comparison with those of the controls (Figure 5A,B).

As MMP reflects activities of electron transport and oxidative phosphorylation, which are required for ATP production, we also measured ATP concentration in brain and liver samples from the cold-treated fish. In agreement with changes in MMP, ATP concentrations in both the brain and liver of the NO and LE fish were significantly lower than those of the controls. Furthermore, ATP concentrations of the NO samples were significantly higher than those of the LE samples (Figure 5C). These data indicate that the ability of Nile tilapia to survive acute cold stress is dependent on the robustness of mitochondrial function.

### 3.5. Cold Stress Induced Expression of Apoptosis-Related Genes

**Figure 6:**
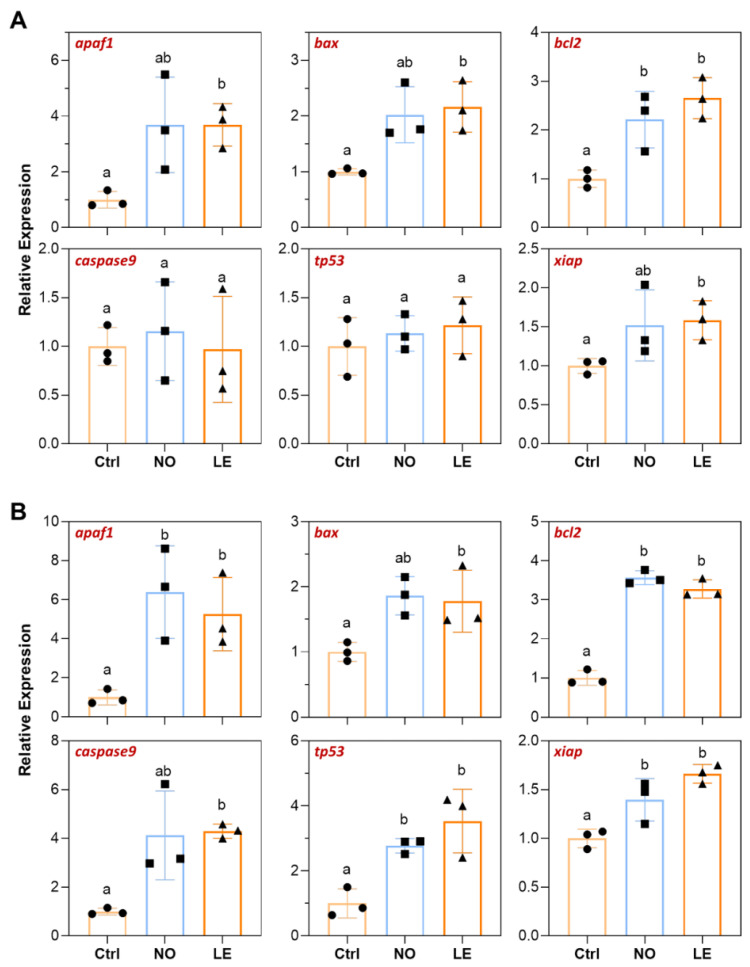
Expression of apoptosis-related genes determined by qPCR. (**A**) Brain. (**B**) Liver. The error bars represent SD (*n* = 3). The dots, squares and triangles represent data of the corresponding experimental groups shown in the horizontal axis. Different letters above the error bars indicate significant difference between the means (*p* ≤ 0.05).

Expressions of genes involved in mitochondria-mediated apoptosis including *apaf1*, *bax*, *bcl2*, *caspase9*, *tp53* and *xiap* in brain and liver of the fish with different ability to withstand acute cold stress were analyzed by qPCR. When compared with the controls, the LE fish demonstrated significant upregulation of *apaf1*, *bax*, *tp53* and *xiap* in brain; however, only *bax* was significantly induced in brains of the NO fish. No significant difference in expression of *bcl2* and *caspase9* was identified among the experimental groups (Figure 6A). For the liver samples, all studied genes were significantly upregulated in the LE fish in comparison with the controls; liver of the NO fish also demonstrated significant induction of all the genes except for *bcl2* and *tp53* (Figure 6B). These results indicate that cold exposure induced expression of apoptosis-related genes in the brain and liver of Nile tilapia. Furthermore, liver for both the NO and LE fish demonstrated more upregulated apoptosis-related genes than the corresponding brain samples. This is consistent with the histological observation that liver is the most sensitive organ to acute cold stress.

### 3.6. Cold Stress Induced Expression of ER Stress Response Genes

**Figure 7:**
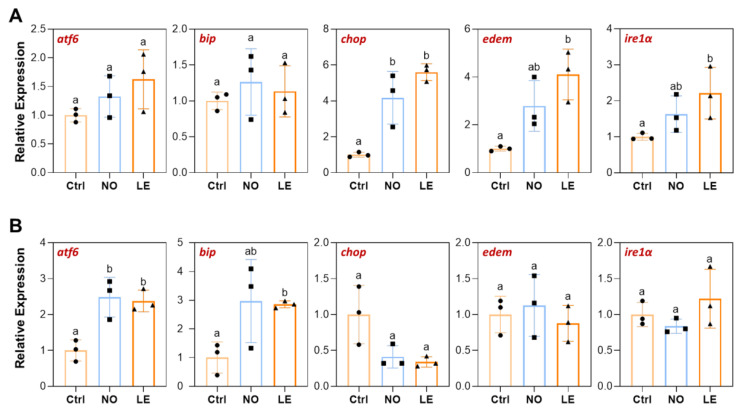
Expression of the ER stress-response-related genes determined by qPCR. (**A**) Brain. (**B**) Liver. The error bars represent SD (*n* = 3). The dots, squares and triangles represent data of the corresponding experimental groups shown in the horizontal axis. Different letters above the error bars indicate significant difference between the means (*p* ≤ 0.05).

Cold stress caused ER structure disturbance in liver of the LE and NO fish (Figure 4A), suggesting that ER stress may be induced. As ER stress can induce mitochondria dysfunction and cell apoptosis [24,25], we measured expression of the ER stress-activated genes including *atf6*, *bip*, *chop*, *edem* and *ire1α* using qPCR to verify that ER stress response was induced by cold stress. In comparison with the controls, the brains of the LE fish demonstrated significant upregulation of *chop*, *edem* and *ire1α*; while brains of the NO group only demonstrated upregulation of *chop* (Figure 7A). Liver of the cold-treated fish (LE and NO) had different upregulated genes compared with brain (Figure 7B). The *bip* and *atf6* genes were upregulated in the LE liver but none of them were differentially expressed among the brain samples (Figure 7A,B). These results indicate that cold exposure induced ER stress response in the brain and liver of Nile tilapia and, again, more prominent changes in expression of the indicator genes were found for the LE samples.

### 3.7. Cold Exposure Resulted in Severe Cell Apoptosis

**Figure 8:**
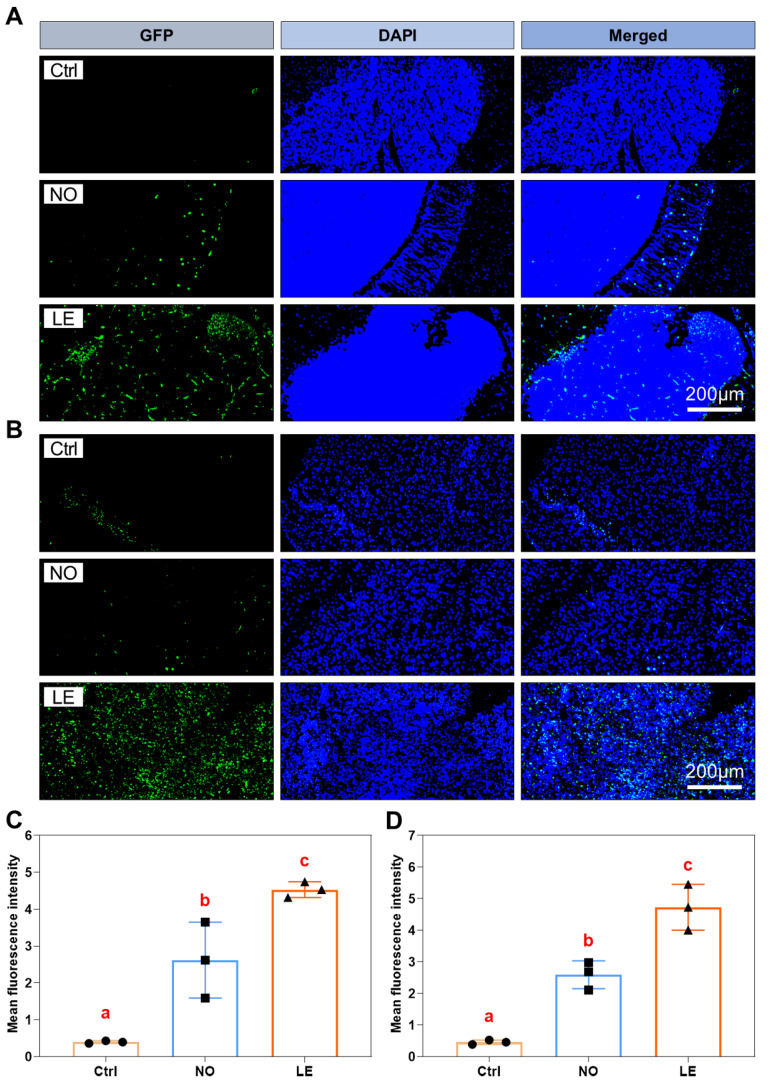
Exposure to acute cold stress resulted in severe cell apoptosis in the brain and liver of Nile tilapia. (**A**,**B**) Photographs indicating apoptotic cells in the brain (**A**) and liver (**B**) sections. Sample numbers for the Ctrl, NO and LE groups are as follows: brain 3, 3, 3; liver 3, 3, 3. (**C**,**D**) Bar charts illustrating intensity of TUNEL signal for the brain (**A**) and liver (**B**) sections. The dots, squares and triangles represent data of the corresponding experimental groups shown in the horizontal axis. The error bars represent SD (*n* = 3). Different letters above the error bars indicate significant difference between the means (*p* ≤ 0.05).

All the above data point to the ultimate fate of cells upon exposure to acute cold stress, namely, apoptosis, the direct cause of cold-induced tissue dysfunction. TUNEL assays were performed to explore the extent of cold-caused cell apoptosis in the brain and liver of the NO and LE fish. The results revealed that the LE fish demonstrated more severe cell apoptosis in both the brain and liver. The NO sections had a markedly lower number of apoptotic cells than the LE ones (Figure 8A,B). Measurements of TUNEL signal (intensity of fluorescence) indicated that the degree of cell apoptosis in both the brain and liver of the LE fish was significantly higher than that of the NO samples (Figure 8C,D). These data demonstrate that exposure to acute cold stress results in severe cell apoptosis in Nile tilapia tissues and the extent of cell apoptosis is intimately related to an individual’s cold resistance.

## 4. Discussion

Cold resistance is an important economic trait of farmed fish. A large number of investigations have been conducted to explore the mechanisms underlying cold susceptibility of tilapia; however, the cellular basis and molecular pathways leading to tissue damage upon cold exposure remain largely unknown. Here, we report the characterization of cold resistance of Nile tilapia, expressed as the survival time of individuals under lethal low-temperature stress (12 °C). Although the roles of genetic factors in determining cold resistance of tilapia have been clearly established [10,15], the effects of body size are inconsistent. No correlation between cold tolerance and fish size (within the range of 23–105 mm standard length) was identified for *Oreochromis mossambicus*, *O. aureus* and their F1 and F2 hybrids [15]. In another study, body size (136 vs. 220 mm) significantly affected mortality of Nile tilapia under cold stress; the smaller fish were less tolerant to low temperature than the larger ones [26]. In our study, no significant correlation was identified between survival time and factors including standard length, body weight and conditional factor. The discrepancy between these results may be ascribed to the distinct physiological status of the experimental fish used for different studies.

During characterization of cold resistance, we found that loss of equilibrium (LE) was the most prominent symptom for the fish injured by cold exposure. Upon suffering the same degree of cold stress (exposure time), the individuals with LE symptom could not revive upon returning to normal temperature, while those that swam normally (NO) could readily resume. These observations indicate that the NO and LE individuals had differential ability to withstand lethal cold stress. Furthermore, this difference cannot be attributed to physiological factors including body size and conditional factor. The fish were exposed to 12 °C low temperature for 72 h and the survivors were classified into the NO and LE groups. The individuals with distinct cold resilience were subjected to histological, biochemical and gene expression assays to explore the cellular and molecular changes underlying cold endurance. The results of histological analysis indicate that exposure to lethal cold stress caused extensive damage to the tissues of Nile tilapia. Cold exposure caused the generation of vacuoles in the brain and liver, atrophy of cardiac and skeletal muscle fibers, collapse and disintegration of gill lamella, shrinkage of renal tubule epithelial cells and reduction of red blood cell numbers in the spleen. These observations shed new light on the cellular changes underlying cold-induced impairments to body functions including motility, metabolism, respiration, osmotic regulation and hematopoiesis.

As the brain is the organ controlling body equilibrium and the liver was found to be more susceptible to cold stress than other organs, they were chosen for the subsequent analyses. Mitochondrial swelling was the most marked subcellular change upon cold exposure for both the brain and liver cells, followed by ER structural disturbance. Measurements of mitochondria size indicate that the LE samples had more severe mitochondria structure disruption than the NO fish. Mitochondrial swelling can be induced by toxicants, calcium overloading and oxidative stress [27,28,29], which reflects the opening of the mitochondrial permeability transition pore and mitochondrial dysfunction [27]. Mitochondrial swelling is reversible and the extent of mitochondrial swelling determines whether the cell can recover from an adverse event [30]. Inability of the LE fish to revive under normal temperature may be ascribed to severe mitochondrial damage that exceeded the reversible point.

Consistently, results of MMP and tissue ATP concentration assays demonstrate that cold exposure significantly decreased cellular MMP and tissue ATP concentration. The LE fish demonstrated significantly lower tissue ATP concentration than the NO ones. These results indicate that exposure to acute cold stress impairs the structure and function of mitochondria and that hardiness of mitochondria is intimately related to an individuals’ cold resistance. Cold-induced damage to mitochondria was also reported for Japanese eel *Anguilla japonica*, where electron microscopy revealed mitochondrial degeneration in hepatocytes [31]. Mitochondrial dysfunction is a major inducer of mitochondria-mediated apoptosis and necrosis, which releases proapoptotic factors such as cytochrome c [32]. Expressions of several genes involved in mitochondria-dependent apoptosis were analyzed by qPCR to connect cold-induced mitochondria dysfunction with cell apoptosis. The results demonstrate that genes such as *apaf1*, *bax* and *tp53* were significantly upregulated in both the brain and liver of the LE fish, while the NO samples demonstrated less upregulated apoptosis-related genes than the LE fish. Our study links mitochondrial dysfunction with the ability of fish to resist cold stress.

ER and mitochondria are physically connected cellular organelles, and induction of ER stress can affect both the morphology and function of mitochondria [25]. A previous study indicated that exposure to cold stress elicited ER stress and activated the intrinsic apoptotic pathway (mitochondria-mediated apoptotic pathway) in zebrafish through disrupting calcium homeostasis [33]. Disturbance of ER structure was observed for liver samples of both the LE and NO fish, suggesting that ER stress may be elicited in Nile tilapia tissues upon cold stress. Upregulation of the ER stress-activated genes such as *atf6*, *bip* and *chop* in the tissues of cold-treated fish confirmed this postulation. Interestingly, upregulation of different ER stress-related genes was found for the brain and liver of the cold-injured Nile tilapia, suggesting that a different branch of ER stress response was activated by cold stress in these two tissues. The molecular mechanisms underlying this phenomenon remain to be investigated.

Finally, as expected from the results of histological, biochemical and gene expression analyses, severe cell apoptosis was identified in tissues of the cold-treated Nile tilapia. Furthermore, cell apoptosis indices for both the brain and liver of the LE fish were significantly higher than those of the NO samples. The degree of cell apoptosis reflects the extent of mitochondria dysfunction and determines whether the fish can survive the cold stress. Taken together, our data indicate that exposure of Nile tilapia to acute cold stress causes ER stress, mitochondria dysfunction, cell apoptosis and systemic tissue damage; the overall adverse effects ultimately result in organism death. We propose that mitochondria dysfunction and cell apoptosis limit the resistance of Nile tilapia to lethal cold stress.

## 5. Conclusions

Median survival time (LT50) of the lab-grown Nile tilapia juveniles upon 12 °C lethal cold exposure was estimated as 3.14 d. Survival time of individuals had no significant correlation with physiological parameters including body size and conditional factor. Cold exposure caused extensive tissue damage to Nile tilapia. Mitochondrial swelling was the most marked subcellular change elicited by cold stress. Consistent with structure disturbance of mitochondria, exposure to acute cold stress resulted in mitochondria dysfunction, upregulation of apoptosis- and ER stress-related genes and severe cell apoptosis. Finally, the extent of the mitochondria-associated cellular and molecular events limits resilience of Nile tilapia to lethal cold stress. Our data provide insights into the cellular and molecular mechanisms underlying cold susceptibility of Nile tilapia.

## Figures and Tables

**Table 1:** Sequence, amplicon size and efficiency of the primers used for qPCR analysis.

Gene Name	Forward 5′-3′	Reverse 5′-3′	Amplicon Size (bp)	Efficiency (%)
Apoptosis-related genes				
*apaf1*	tggccagtttgctccatagg	tgacaaacactacgggcctc	135	102.6
*bcl2*	gtgggcaggatcgtagagtg	ctgagacctccggctttcag	135	98.2
*bax*	tactttgcatgccgactcgt	caccttgctccctgatccag	127	100.7
*caspase9*	gttgtccgccctgtaatcca	gtcttaactgccacccgtca	124	95.3
*tp53*	ggcagggaccgtaaaactga	tttgtggacgtgtcaggagg	104	104.4
*xiap*	cttgtgtttccagtgcggtg	ttgccaaaaggaagctgcac	104	106.8
ER stress-related genes				
*atf6*	atgagctggagaaactcggc	atctggaacaccgttggcat	92	101.6
*bip*	ttcaagaatggacgcgtgga	caccaatcagacgctcacct	100	107.6
*chop*	cccctacatgcaccgagaag	gacaccgtcaccccatgtc	101	103.4
*edem*	tggacactctgttggtgctc	tgcctcaaaaacttgcacagt	110	90.4
*ire1α*	ccagttcttcaggtccccac	gccttcattgttcttcccgc	99	104.3
Internal references				
*ef1a*	tcagatcgctgcaggctatg	ggcatctccggacttgacaa	145	107.7
*gapdh*	tgacgctcccatgtttgtca	cagttggttgtgcaggaagc	90	103.3

## Data Availability

The data presented in this study are available in the article.
